# VOLUNTEERING OF FORMER DEMENTIA CAREGIVERS: A PILOT INTERVENTION STUDY IN THE CHINESE AMERICAN COMMUNITY

**DOI:** 10.1093/geroni/igae098.0103

**Published:** 2024-12-31

**Authors:** Jinyu Liu, Yifan Lou, Ethan Siu Leung Cheung

**Affiliations:** Baylor University, Waco, Texas, United States; Virginia Commonwealth University, New Haven, Virginia, United States; University of Utah, Salt Lake City, Utah, United States

## Abstract

To promote social engagement of elders of color and mental health of caregivers, we developed a three-month peer mentoring program (PMP) to train and supervise older Chinese Americans who used to be family caregivers of people with dementia (former caregivers) to provide weekly one-on-one mentoring to current caregivers in the same ethnic community. This pilot study aims to test the feasibility and preliminary effect of the PMP. A pilot randomized control trial was conducted among 38 current caregivers and 8 former caregivers in New York City. The current caregivers were randomly assigned to the PMP and control groups. Quantitative and qualitative data were collected at baseline, 3-month, and 9-month from former and current caregivers. T-test and difference-in-difference analyses were used to analyze quantitative data. A thematic analysis was conducted to understand former caregivers’ volunteering experience. The feasibility of the intervention was high, as indicated by an acceptable retention rate, fidelity, and positive feedback from former caregivers. Compared with the control group, the current caregivers in the PMP group had greater reductions in scores for loneliness, caregiver burden and depressive symptoms. Though former caregivers did not report significant changed depressive symptoms and loneliness after participating in the PMP due to their minimal symptoms at baseline, they highlighted that the intervention enhanced their sense of purpose, fulfillment, growth, and self-efficacy in narrative responses. The results indicated the feasibility and efficacy of empowering existing human resources of former caregivers in the same ethnic community to improve the mental health of Chinese caregivers.

